# Responding, fast and slow: Visual detection and localization performance is unaffected by retrieval

**DOI:** 10.3758/s13414-023-02810-5

**Published:** 2023-11-20

**Authors:** Lars-Michael Schöpper, Christian Frings

**Affiliations:** 1 Department of Cognitive Psychology, University of Trier, 54286 Trier, Germany; 2 Institute for Cognitive and Affective Neuroscience (ICAN), University of Trier, Trier, Germany

**Keywords:** Action control, Attentional orienting, Perception, Task demands

## Abstract

According to action control theories, responding to a stimulus leads to the binding of the response and stimulus features into an event file. Repeating any component of the latter retrieves previous information, affecting ongoing performance. Based on years of attentional orienting research, recent boundaries of such binding theories have been proposed as binding effects are fully absent in visual detection (e.g., Schöpper et al., 2020, *Attention, Perception, & Psychophysics, 82*(4), 2085–2097) and localization (e.g., Schöpper & Frings, 2022; *Visual Cognition, 30*(10), 641–658) performance. While this can be attributed to specific task demands, the possibility remains that retrieval of previous event files is hampered in such tasks due to overall fast responding. In the current study we instructed participants to signal the detection (Experiment 1) and location (Experiment 2) of dots orthogonally repeating or changing their nonspatial identity and location. Crucially, the dots were either hard or easy to perceive. As expected, making targets hard to perceive drastically slowed down detection and localization response speed. Importantly, binding effects were absent irrespective of perceptibility. In contrast, discriminating the nonspatial identity of targets (Experiment 3) showed strong binding effects. These results highlight the impact of task-dependence for binding approaches in action control.

## Introduction

Throughout﻿﻿ the day, we constantly interact with our surroundings—may it be turning on the light switch, grabbing the coffee mug, or ringing the bicycle bell while riding to work. According to action control theories, such bodily movements constitute actions because they are done with an intention or anticipated goal in mind (Frings et al., 2020; Prinz, 1998). Several theories have been developed to describe how such actions are accomplished. According to the theory of event coding (Hommel et al., 2001), responding to a stimulus (like a light switch or a key press in a laboratory setting) leads to the integration of stimulus information and the response into a so-called event file (Hommel, 2004). If now any component of the event file repeats, the previous information is retrieved: This causes benefits for full repetitions, but interference if information does not fully match—partial repetition costs arise (Hommel, 1998). These lead to so-called stimulus–response (S-R) binding effects (Hommel, 1998), and even include the coupling of response-irrelevant information (Frings et al., 2007). These S-R binding effects can be measured in prime-probe sequences, in which participants respond to a stimulus in a prime display followed by a response to a stimulus in a probe display. From prime to probe, a response-irrelevant feature, for example, flanking letters (Frings et al., 2007), color (Schöpper & Frings, 2022), or location (Schöpper et al., 2020), is orthogonally varied with response repetitions and changes. In these designs binding effects can be reliably observed. However, following Frings et al. (2020), the dissociable processes of binding and retrieval are not only present in such prime–probe sequences but are active in numerous experimental designs with sequential structure, like, for example, priming (Henson et al., 2014), conflict tasks (Davelaar & Stevens, 2009), or task switching (Koch et al., 2018).

S-R binding effects or more general binding and retrieval are tacitly assumed to underlie all actions (e.g., Frings et al., 2020; Hommel, 2004). However, recently, important boundaries of binding effects have been proposed. While referring to years of attentional orienting research (for an overview, see Huffman et al., 2018), S-R binding effects are typically not observed in visual detection (Schöpper et al., 2020) or localization (Hilchey et al., 2018; Schöpper & Frings, 2022; Schöpper et al., 2022a) performance. Here, inhibition of return (IOR; Posner et al., 1985; for a review see, e.g., Klein, 2000)—that is, an overall cost for location repetitions—is the main pattern. So far, this seems to be modality-dependent, as S-R binding can be observed in auditory detection performance, where a repeated detection response benefits from repeating the pitch of a sound (Mondor & Leboe, 2008; Schöpper & Frings, 2023).

In some cases, IOR can be modulated by nonspatial features: A benefit for nonspatial feature changes can arise (Law et al., 1995), especially at location repetitions (Hu et al., 2011, 2013). While mostly investigated with cue–target sequences (i.e., responding to the second of two sequentially presented stimuli), this effect, typically referred to as nonspatial IOR (see, however, Fox & De Fockert, 2001, for arguing that this effect can be better explained by repetition blindness), can be observed when sequentially responding to every target. For example, Chao et al. (2020) investigated if there is time-dependent memory retrieval (Tulving & Thomson, 1973) for IOR. They presented two to-be-detected targets in a sequence while repeating or changing their color (in their study treated as “color context”) and found larger IOR if the target repeated its color (Experiment 1), which increased with increasing response times (Experiment 2). In the latter case, a color change benefit emerged at location repetitions (cf. Hu et al., 2011; see also direct localization responses of Experiment 1 in Schöpper et al., 2022a). In general, nonspatial IOR often emerges in complex designs (Hu et al., 2013) and/or sometimes at especially late responses (e.g., Chao et al., 2020; Schöpper et al., 2022a) and is often explained by a detection cost for perceptually similar targets at the same location (Lupiáñez, 2010; Lupiáñez et al., 2013): If a cue with nonspatial identity (e.g., color or shape) appears at a certain location, a following target with the same nonspatial identity appearing at the exact same location might be “absorbed” by the previous activation of the cue—a detection cost for the target occurs. If, however, the target has a different nonspatial identity to the cue, this detection cost is circumvented as the target is perceived as being different (for a discussion, see Hu et al., 2011). Importantly, this nonspatial IOR effect—an overall change benefit or a partial repetition benefit at location repetitions—is opposite to what binding approaches would expect (for a discussion, see Schöpper & Frings, 2022; Schöpper et al., 2022a).

Several ideas have been proposed why S-R binding effects are regularly absent in detection (and localization) performance. First, it has been argued that a discriminatory component is what leads to the observation of S-R binding effects (e.g., Schöpper et al., 2020, 2022a, b)—if it is absent, binding effects are not observed. This fits well with different outcomes for detection and discrimination procedures in visual search (e.g., Krummenacher et al., 2009; Müller & Krummenacher, 2006; for a discussion, see Schöpper et al., 2020), highlighting the importance of a discrimination response for observing partial repetition costs in search displays (Zehetleitner et al., 2012). Second, it has been argued that a lack of attention towards the nonspatial feature of the target stimulus leads to such null effects (Huffman et al., 2018, 2020). In fact, attention has been found to have a modulating role on the occurrence of binding and retrieval (Moeller & Frings, 2014; Singh et al., 2018), and processing spatial information more strongly prior responding can lead to binding effects in detection (Hilchey et al., 2020) and localization (Schöpper et al., 2022a) procedures (cf. Hommel, 2007). Third, detection and localization performance is typically way faster than discrimination performance (e.g., Pratt & Castel, 2001); the “horserace account” (Frings & Moeller, 2012; see Neill, 1997) argues that with target onset two processes—response generation and retrieval—start, which compete until the response is executed. Following this, it can be argued that the easily computed detection or localization response is simply executed so fast that retrieval has no chance to alter it (see Schöpper et al., 2020).

Following the horserace account, Schöpper et al. (2020) argued that S-R binding effects might not occur if a response is executed too fast. In turn, slowing down overall responding in detection and localization performance should spur on retrieval. Making stimuli hard to identify until fixated (Hilchey et al., 2018) or making them less salient (Töllner et al., 2011) slows down manual responding. Referring to such perceptual effects, we instructed participants to respond to visual targets that were either easy or hard to perceive.

## Current study

We instructed participants to respond to target dots that were either red or blue on black background (easy perceptibility) or light grey or dark grey on intermediate grey background (hard perceptibility). Targets could orthogonally vary their location and color. Participants had to signal the detection of the dots (Experiment 1), localize them (Experiment 2), or discriminate their color (Experiment 3). In typical discrimination tasks investigating S-R binding (e.g., Frings et al., 2007; Schöpper et al., 2020), a binding pattern can be derived from the interaction of task-irrelevant feature and response, in that participants should be slower in partial repetitions compared with full repetitions and full changes. In detection performance as used in Experiment 1 of the current study, a binding pattern could be derived from an interaction of two varying features (color–location binding irrespective of the response) as well. However, as every response is a response repetition (i.e., the space bar is pressed for every target), a binding effect could manifest as a benefit of location repetition (location–response binding), or a benefit of color repetition (color–response binding): Pressing the space bar in the prime display should result in binding of a feature (i.e., location or color) to the detection response, which should be retrieved if the feature (i.e., the location or color) repeats in the probe display. Previously, all these predictions of possible outcomes of binding and retrieval affecting visual detection performance have been falsified and simply IOR has been found (Schöpper & Frings, 2023; Schöpper et al., 2020). In localization (Experiment 2) and discrimination (Experiment 3) performance, a binding effect can be derived from the interaction of color and location depicting partial repetition costs, resulting from a binding pattern between localization response × color (Experiment 2) and color response × location (Experiment 3). Main effects of color and location would be orthogonal to repeating or changing the response and thus would not be an indicator for retrieval in the sense of binding effects. Yet, binding effects are—as in visual detection performance—typically not observed in localization procedures (Schöpper & Frings, 2022), while discrimination tasks lead to reliable effects (Schöpper et al., 2020). If overall fast responding is the reason for absent retrieval in detection and localization performance, an experiment with slowed down responses should evoke a binding pattern caused by any of the possible binding possibilities. However, if no effect is observed, the previously set boundary of binding theories is shielded from a time-based explanation, supporting the view of task dependency for binding approaches in action control (Schöpper & Frings, 2022; Schöpper et al., 2020).

## Experiment 1

### Methods

#### Participants

IOR is a stable effect coming with medium to high effect sizes (e.g., *d* = 1.14 in Schöpper & Frings, 2022). A binding pattern in visual detection performance is usually absent (Huffman et al., 2018; Schöpper et al., 2020). The sample size geared to that in Schöpper et al. (2020). In turn, 30 students of the University of Trier participated for either course credit or a monetary reward (10 Euro). The experiment was conducted in accordance with ethical guidelines of the University of Trier. All participants gave written informed consent. One participant reported an uncorrected color blindness; however, the data were inconspicuous when compared with the sample and thus included in the analysis. All others reported normal or corrected-to-normal vision. One participant was a heavy outlier in errors made (high number of missed responses) when compared with the sample and was excluded from analysis. Under α = 0.05 (one-tailed), the final sample of 29 participants (24 female, four male, one other; *M*_Age_ = 23.07 years, *SD*_Age_ = 3.04; age range: 20—32 years) yields a power of 1 − β = 0.99 for detecting a large IOR effect of at least *d* = 0.08 (G*Power, Version 3.1.9.2; Faul et al., 2007).

#### Apparatus and materials

The experiment geared to the detection task used in Schöpper et al. (2020). The experiment was programmed in E-Prime 2.0 and was presented on a monitor with a display resolution of 1,680 × 1,050 px (length × height: 44.45° × 28.63° of visual angle derived from a distance of 58 cm; however, we did not use a chin rest, so perceived sizes may have varied). A white (R/G/B: 255/255/255) fixation cross (0.40° × 0.40°) was presented in the left screen half on a black (R/G/B: 0/0/0) or grey (R/G/B: 128/128/128) background. Targets were circles 0.69° in diameter and appeared in the right screen half, 11.13° away from the fixation cross on the *x*-axis and 3.06° above or below it. In catch trials, targets were invisible on the grey or black background. Targets were red (R/G/B: 224/32/64), blue (R/G/B: 64/64/192), light grey (R/G/B: 134/134/134), or dark grey (R/G/B: 122/122/122).

#### Design

The experiment used a 2 (perceptibility: easy vs. hard) × 2 (location relation: repetition vs. change) × 2 (nonspatial feature relation: repetition vs. change) design. All variables were varied within subjects. Binding effects in detection performance can be either derived from main effects of location or nonspatial feature with benefits for repetitions, or from an interaction of location and nonspatial feature.

#### Procedure

Participants worked through the easy perceptibility block followed by the hard perceptibility block, or the hard perceptibility block followed by the easy perceptibility block. Order was alternated with every participant. Each block started with instructions, followed by practice trials and experimental trials.

**Fig. 1:**
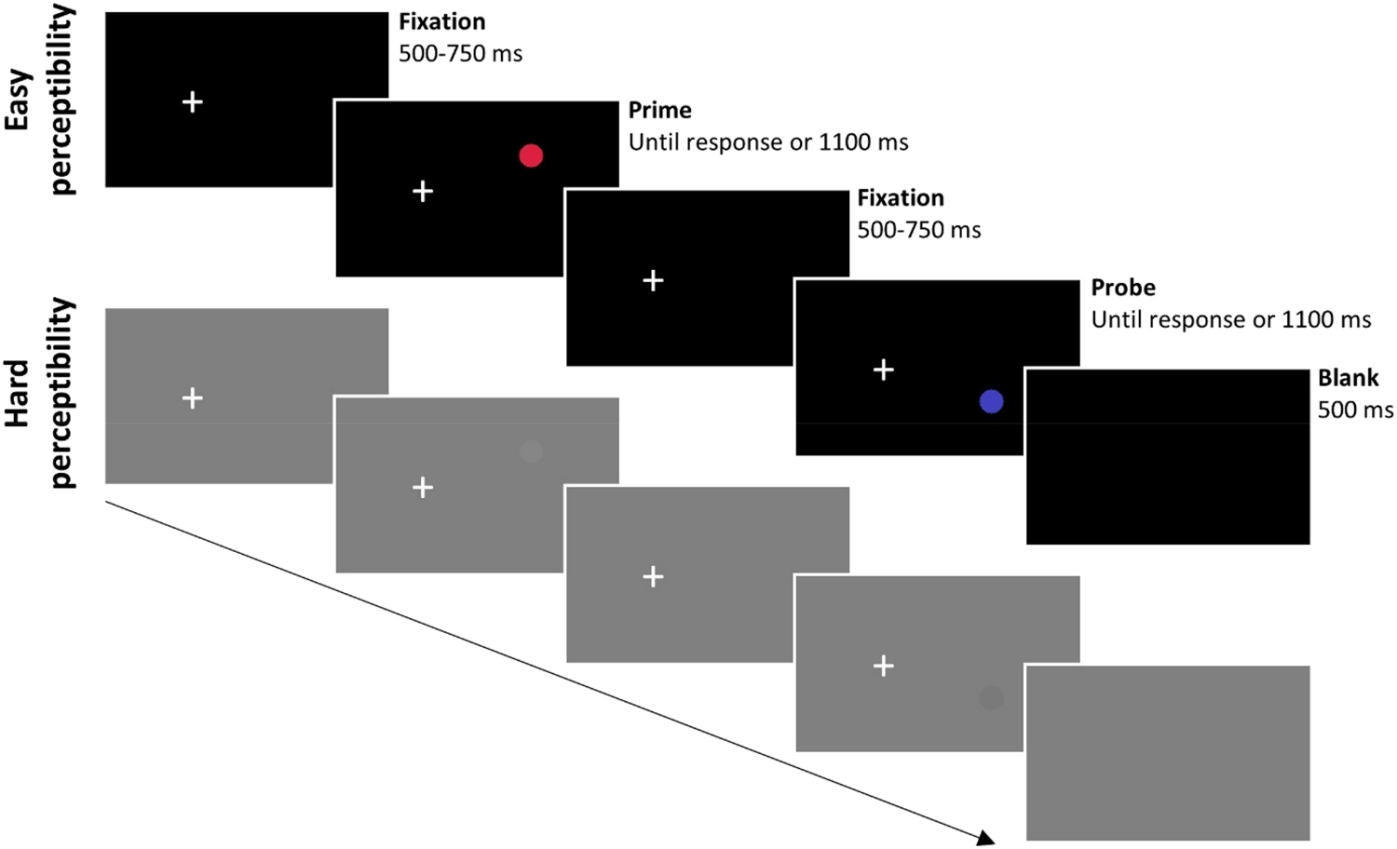
Example prime-probe sequences of Experiment 1 and 3, depicting trials with location change and color change in the easy (top row) and hard (bottom row) perceptibility block (not drawn to scale). Experiment 2 was identical, except for the fixation cross being presented at center and targets appearing left or right of fixation. (Color figure online)

Targets were presented in a prime-probe structure. Participants first saw a prime target and gave a response to it, followed by a probe target and a response to it. A trial started with the fixation cross appearing on the left. This fixation cross remained for the whole sequence until probe response execution and participant were instructed to fixate throughout. After a variable interval of 500–750 ms, the prime stimulus appeared on the right side of the screen at either the top or bottom position. In the hard block the target could be light grey or dark grey on an intermediate grey background, and in the easy block the target could be red or blue on a black background. Participants were instructed to press the space bar (with the right index finger) upon detection of the target; they were told to signal the detection of a dot irrespective of its color or location. Targets remained visible upon response or until 1,100 ms had passed. After the prime display, the fixation cross was shown in isolation, again for an interval of 500–750 ms, and then accompanied by the probe target, to which a detection response had to be made as outlined for the prime display. After the probe display, the display turned blank for 500 ms, ending a prime–probe sequence (see Fig. 1). If participants failed to press the space bar in the time window during the prime or probe display, an error message appeared directly after for 1,500 ms. In some trials no target appeared either during the prime display or during the probe display—that is, a catch trial. Here, participants simply had to wait in the respective display.

From prime to probe the location of the target could repeat (location repetition [LR]) or change (location change [LC]), and the color of the target could repeat (color repetition [CR]) or change (color change [CC]) in each perceptibility block. There were catch trials in which either the prime or probe target was absent. All location and color combinations were pseudorandomly balanced, and conditions were drawn randomly. In each perceptibility block the experiment started with eight practice trials drawn randomly from the set of all different combinations, and participants received feedback after every response (or nonresponse in case of catch trials). This was followed by the experiment with 64 trials for every condition (i.e., 256 trials in total), 32 prime-catch trials, and 32 probe-catch trials, in which participants received feedback only for incorrect responses. After every 80th experimental trial, as well as between blocks, participants could take a self-paced break.

### Results

Reaction times above 50 ms or below 3 interquartile range above the third quartile of a participant’s distribution (Tukey, 1977) were included for analysis. Additionally, we only included trials in which both prime and probe response were correct (i.e., no missed responses). This led to 1.83% of trials being discarded. Catch trials were not analyzed.

A 2 (perceptibility: easy vs. hard) × 2 (location relation: repetition vs. change) × 2 (nonspatial feature relation: repetition vs. change) repeated-measures ANOVA on probe reaction times (see Table 1) revealed a main effect of perceptibility, *F*(1, 28) = 127.46, *p* < .001, ${\upeta }_{p}^{2}$ = 0.82. This effect was in the expected direction in that participant were faster in the easy perceptibility block (307 ms) compared with the hard perceptibility block (372 ms). There was a main effect of location relation, *F*(1, 28) = 22.52, *p* < .001, ${\upeta }_{p}^{2}$ = 0.45, showing a cost for location repetition (345 ms) over location change (334 ms)—that is, IOR. Yet there was no main effect of color relation, *F*(1, 28) = 0.02, *p* = .887, ${\upeta }_{p}^{2}$ < 0.01, no interaction of color relation and location relation, *F*(1, 28) = 1.52, *p* = .227, ${\upeta }_{p}^{2}$ = 0.05, nor were any of these effects modulated by perceptibility (all *F*s ≤ 0.23, all *p*s ≥ .637).

**Table 1:** Mean reaction times in ms

	Experiment 1: Detection task			
	Easy perceptibility		Hard perceptibility	
	Color Repetition	Color Change	Color Repetition	Color Change
Location Repetition	312	311	379	376
Location Change	301	302	365	367
	Experiment 2: Localization task			
	Easy perceptibility		Hard perceptibility	
	Color Repetition	Color Change	Color Repetition	Color Change
Location Repetition	322	321	350	355
Location Change	314	313	339	343
	Experiment 3: Discrimination task			
	Easy perceptibility		Hard perceptibility	
	Color Repetition	Color Change	Color Repetition	Color Change
Location Repetition	410	465	466	521
Location Change	433	432	489	492

**Fig. 2:**
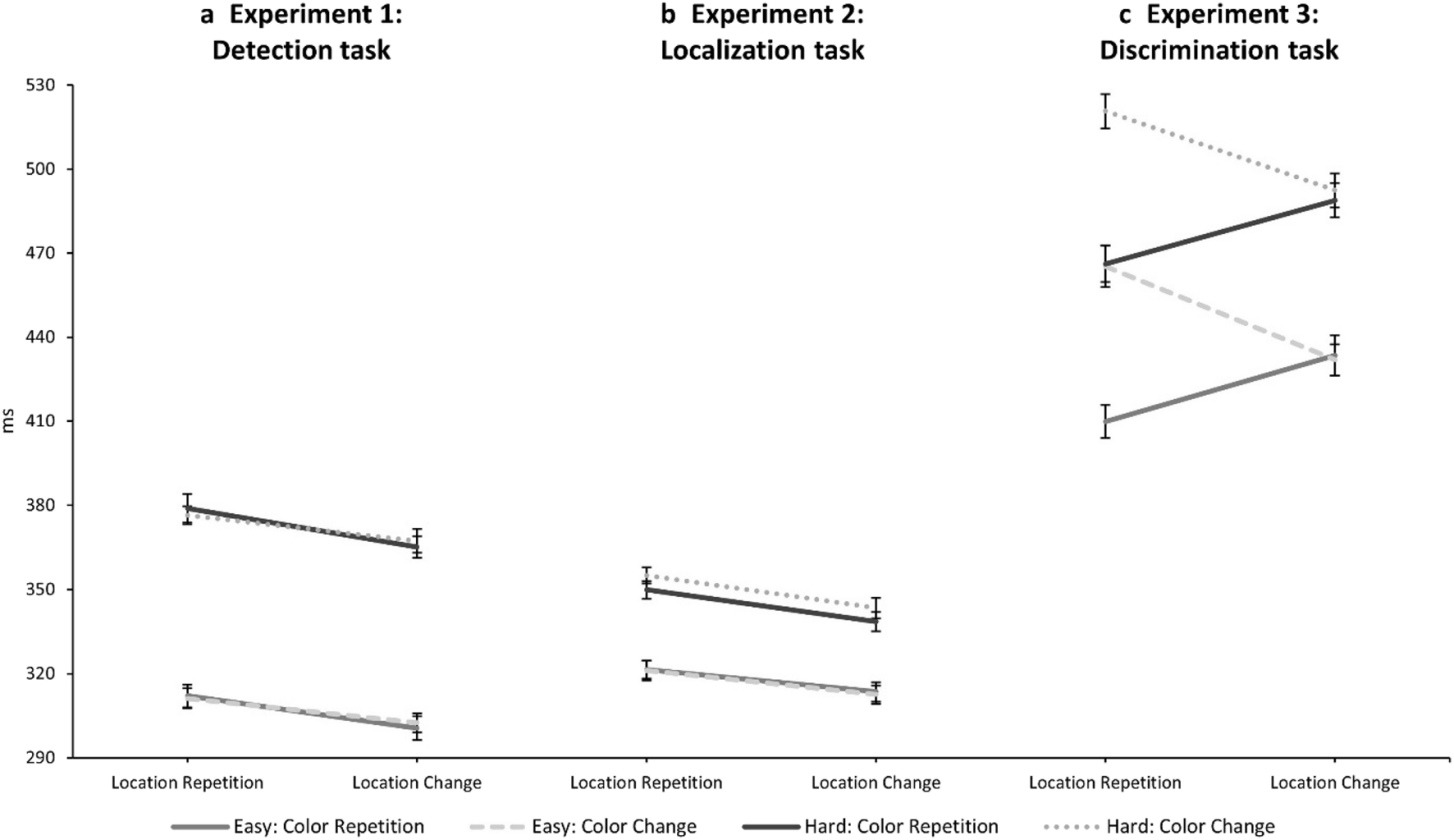
Interactions of location relation and color relation separate for the easy and hard perceptibility block in **a**. Experiment 1 (detection task), **b**. Experiment 2 (localization task), and **c.** Experiment 3 (discrimination task). Error bars represent within-subject standard error after Cousineau–Morey (Cousineau, 2005; Morey, 2008)

For sake of completeness, we looked at the individual perceptibility blocks separately. In the easy perceptibility block, only the main effect of location relation was significant, *F*(1, 28) = 19.53, *p* < .001, ${\upeta }_{p}^{2}$ = 0.41, depicting IOR (LR: 312 ms; LC: 301 ms). Yet the main effect of color relation, *F*(1, 28) = 0.12, *p* = .737, ${\upeta }_{p}^{2}$ < 0.01, and the interaction of color relation and location relation,* F*(1, 28) = 0.59, *p* = .449, ${\upeta }_{p}^{2}$ = 0.02, were not significant. In the hard perceptibility block, only the main effect of location relation was significant, *F*(1, 28) = 13.20, *p* = .001, ${\upeta }_{p}^{2}$ = 0.32, also depicting IOR (LR: 378 ms; LC: 366 ms). Again, the main effect of color relation, *F*(1, 28) < 0.01, *p* = .953, ${\upeta }_{p}^{2}$ = 0.00, and the interaction of color relation and location relation,* F*(1, 28) = 1.69, *p* = .205, ${\upeta }_{p}^{2}$ = 0.06, were not significant. The data patterns are presented in Fig. 2a.

#### Error rates

Error rate is the percentage of incorrect (i.e., missed) probe responses after correct prime responses. We thus excluded all incorrect (i.e., missed) prime responses (0.28%). Participants barely missed a response; thus, error rates were close to ceiling. However, percentage of missed probe responses after given prime responses was higher in the hard perceptibility block (0.48%) compared with the easy perceptibility block (0.18%), *t*(28) = 3.35, *p* = .002, *d* = 0.62.

### Discussion

In Experiment 1, participants signaled the detection of target dots which were either red or blue on black background—assumed to be easily perceptible—or light or dark grey on intermediate grey background—thus assumed to be harder to perceive. Participants were slower in detecting grey targets, confirming that perceptibility was impeded in this condition. We observed IOR (e.g., Klein, 2000)—that is, a location repetition cost—in both perceptibility blocks. Yet neither did we observe a main effect of color relation nor any interaction between color and location relation. Importantly, this did not hinge on the perceptibility of targets. Visual detection performance was completely unaffected by repeating or changing nonspatial features.

## Experiment 2

Detection and localization procedures have in common that effects of binding and retrieval in the sense of stimulus–response binding effects are typically absent (Huffman et al., 2018; Schöpper & Frings, 2022; Schöpper et al., 2020). However, localization responses are typically slower compared with detection procedures (e.g., Pratt & Castel, 2001). We thus thought it prudent to replicate the absence of binding effects in even slower localization responses. Thus, Experiment 2 used the same design as Experiment 1, except that participants were asked to localize the target dots.

### Methods

#### Participants

Sample size in Experiment 2 followed the same considerations as Experiment 1. In turn, 30 students of the University of Trier participated for either course credit or a monetary reward (10 Euro). One participant reported an uncorrected visual impairment; however, the data were inconspicuous when compared with the sample and thus included in the analysis. All others reported normal or corrected-to-normal vision. Two participants were heavy outliers in errors made (high number of missed responses and/or incorrect responses) when compared with the sample and excluded from analysis. Under α = 0.05 (one-tailed), the final sample of 28 participants (23 female, five male; *M*_Age_ = 24.14 years, *SD*_Age_ = 4.12; age range: 19–33 years) yields a power of 1 − β = 0.99 for detecting a large IOR effect of at least *d* = 0.08 (G*Power, Version 3.1.9.2; Faul et al., 2007).

#### Apparatus, materials, design, and procedure

Experiment 2 was identical to Experiment 1, except for the following. The fixation cross was presented at center. Instead of appearing at an upper or lower position in the right hemisphere, target stimuli appeared 5.57° to the left or right of the fixation cross and in the same vertical plane as the latter. We did so because localizing spatially incompatible targets—which would be the case when giving left/right responses for targets appearing at the top or bottom of a screen as used in Experiment 1—can spur on binding effects (Geissler et al., 2023; Schöpper et al., 2022a). Participants were instructed to press the F key for left targets and the J key for right targets. With this design, the location relation factor becomes response relevant and completely confounded with response repetitions and changes (cf. Schöpper & Frings, 2022).

### Results

We used the same cut-off criteria as reported for Experiment 1. This led to 3.79% of trials being discarded.

A 2 (perceptibility: easy vs. hard) × 2 (location relation: repetition vs. change) × 2 (nonspatial feature relation: repetition vs. change) repeated-measures ANOVA on probe reaction times (see Table 1) revealed a main effect of perceptibility, *F*(1, 27) = 61.52, *p* < .001, ${\upeta }_{p}^{2}$ = 0.70 (easy perceptibility block: 317 ms; hard perceptibility block: 347 ms). There was a main effect of location relation, *F*(1, 27) = 7.32, *p* = .012, ${\upeta }_{p}^{2}$ = 0.21, showing a cost for repeating (337 ms) compared with changing (327 ms) the response-relevant location feature—that is, IOR. There was no main effect of color relation, *F*(1, 27) = 2.75, *p* = .109, ${\upeta }_{p}^{2}$ = 0.09, which, however, was further modulated by perceptibility, *F*(1, 27) = 5.09, *p* = .032, ${\upeta }_{p}^{2}$ = 0.16: In the hard perceptibility block, repeating the color was faster (344 ms) compared with changing it (349 ms); in contrast, this color repetition benefit was absent in the easy perceptibility block (CR: 318 ms; CC: 317 ms). As with Experiment 1, the interaction of color relation and location relation was not significant, *F*(1, 27) = 0.06, *p* = .803, ${\upeta }_{p}^{2}$ < 0.01, nor were any of these effects modulated by perceptibility (all *F*s ≤ 1.69, all *p*s ≥ .204).

In the easy perceptibility block, only the main effect of location relation was significant, *F*(1, 27) = 4.26, *p* = .049, ${\upeta }_{p}^{2}$ = 0.14, depicting IOR (LR: 321 ms; LC: 313 ms). However, the main effect of color relation, *F*(1, 27) = 0.14, *p* = .708, ${\upeta }_{p}^{2}$ = 0.01, and the interaction of color relation and location relation,* F*(1, 27) = 0.06, *p* = .816, ${\upeta }_{p}^{2}$ < 0.01, were not significant. In the hard perceptibility block, the main effect of location relation was significant, *F*(1, 27) = 9.73, *p* = .004, ${\upeta }_{p}^{2}$ = 0.27, also depicting IOR (LR: 352 ms; LC: 341 ms). Additionally, the main effect of color relation was significant, *F*(1, 27) = 7.40, *p* = .011, ${\upeta }_{p}^{2}$ = 0.22, depicting the abovementioned color repetition benefit (CR: 344 ms; CC: 349 ms). The interaction of color relation and location relation was not significant, *F*(1, 27) < 0.01, *p* = .955, ${\upeta }_{p}^{2}$ = 0.00. The data patterns are presented in Fig. 2b.

#### Error rates

As with Experiment 1, participants barely made errors (missing a response or pressing an incorrect key); thus, error rates were close to ceiling. We excluded all incorrect prime responses (1.51%). Percentage of probe errors after correct prime responses was descriptively higher in the hard perceptibility block (1.27%) compared with the easy perceptibility block (0.98%), which, however, was not significant, *t*(27) = 0.29, *p* = .181, *d* = 0.26.

### Discussion

In Experiment 2, participants localized target dots as being left or right to the fixation cross. Again, we observed IOR and no binding pattern between location relation and color relation in both perceptibility blocks. Interestingly, in the hard perceptibility block we observed a color (i.e., greyscale) repetition benefit (e.g., Becker & Horstmann, 2009) sharing similarities with priming-of-pop-out effects (e.g., Maljkovic & Nakayama, 1994; Moher & Song, 2014). This suggests that this feature repetition benefit either emerges with time or it emerges if detectability is impeded and a repetition benefits search efficiency (cf. Becker & Ansorge, 2013). Importantly, this effect was completely independent from repeating or changing the location/response (cf. Schöpper et al., 2023).

## Experiment 3

In two experiments we showed that stimulus–response binding effects do not occur in detection and localization procedures even if perceptibility is impaired. One might muse if the greyscale dots in the latter led to an insufficient perception of these as being of different identity (note, however, that we observed a color repetition benefit in Experiment 2). We decided to conduct a third experiment in which we used the same design as Experiment 1 except that participants were asked to discriminate the color of the target dots. We expected binding effects to occur in both perceptibility blocks.

### Methods

#### Participants

Binding effects between response and location can be very strong (e.g., *d* > 2.5 in Schöpper et al., 2020). For finding an effect with such a size (*d* = 2.5), five participants are sufficient (α = 0.05, one-tailed; 1 − β = 0.99; G*Power, Version 3.1.9.2; Faul et al., 2007). However, we decided to increase the sample size to potentially find a modulation by perceptibility. In turn, 16 students of the University of Trier participated for either course credit or a monetary reward (10 Euro). Two participants reported a color blindness; one was a mild outlier in incorrect responses to the prime display, the other one was inconspicuous when compared with the sample. We included both in the analysis, as none was an outlier in probe responses. All others reported normal or corrected-to-normal vision. Under α = 0.05 (one-tailed) the final sample of 16 participants (11 female, five male; *M*_Age_ = 24.19 years, *SD*_Age_ = 3.33; age range: 20–32 years) yields a power of 1 − β = 1.00 for detecting a large binding effect of at least *d* = 2.5 (G*Power, Version 3.1.9.2; Faul et al., 2007).

#### Apparatus, materials, design, and procedure

Experiment 3 was identical to Experiment 1 except for the following: Participants were instructed to press the F key for blue targets and the J key for red targets in the easy perceptibility block, and the F key for light grey targets and the J key for dark grey targets in the hard perceptibility block. With this design, the color relation factor becomes response-relevant and completely confounded with response repetitions and changes (cf. Schöpper & Frings, 2022; Schöpper et al., 2020).

### Results

We used the same cut-off criteria as reported for Experiment 1. This led to 9.73% of trials being discarded.

A 2 (perceptibility: easy vs. hard) × 2 (location relation: repetition vs. change) × 2 (nonspatial feature relation: repetition vs. change) repeated-measures ANOVA on probe reaction times (see Table 1) revealed a main effect of perceptibility, *F*(1, 15) = 69.95, *p* < .001, ${\upeta }_{p}^{2}$ = 0.82 (easy perceptibility block: 435 ms; hard perceptibility block: 492 ms). There was no main effect of location relation, *F*(1, 15) = 1.04, *p* = .324, ${\upeta }_{p}^{2}$ = 0.07. There was a main effect of color relation, *F*(1, 15) = 24.34, *p* < .001, ${\upeta }_{p}^{2}$ = 0.62, showing that color response repetitions were faster (450 ms) compared with changes (478 ms). Crucially, the interaction of color relation and location relation was significant, *F*(1, 15) = 75.77, *p* < .001, ${\upeta }_{p}^{2}$ = 0.84: When the color response repeated, participants were faster when the location repeated (438 ms) compared with changed (461 ms), which was significant when tested against each other, *t*(15) = 4.18, *p* < .001, *d* = 1.05. In contrast, when the color response changed, participants were faster when the location changed (462 ms) compared with repeated (493 ms), which was significant when tested against each other, *t*(15) = 7.37, *p* < .001, *d* = 1.84. This was not further modulated by perceptibility, *F*(1, 15) = 0.55, *p* = .468, ${\upeta }_{p}^{2}$ = 0.04, nor were any of the other effects modulated by perceptibility (all *F*s ≤ 1.70, all *p*s ≥ .686).

In the easy perceptibility block, the main effect of color relation was significant, *F*(1, 15) = 14.07, *p* = .002, ${\upeta }_{p}^{2}$ = 0.48 (CR: 422 ms; CC: 449 ms). The main effect of location relation was not significant, *F*(1, 15) = 0.95, *p* = .346, ${\upeta }_{p}^{2}$ = 0.06. The interaction of color relation and location relation was significant, *F*(1, 15) = 95.07, *p* < .001, ${\upeta }_{p}^{2}$ = 0.86 (CRLR: 410 ms; CRLC: 433 ms; CCLR: 465 ms; CCLC: 432 ms). In the hard perceptibility block, the main effect of color relation was significant as well, *F*(1, 15) = 23.04, *p* < .001, ${\upeta }_{p}^{2}$ = 0.61 (CR: 478 ms; CC: 507 ms). The main effect of location relation was not significant, *F*(1, 15) = 0.48, *p* = .497, ${\upeta }_{p}^{2}$ = 0.03. The interaction of color relation and location relation was significant, *F*(1, 15) = 34.27, *p* < .001, ${\upeta }_{p}^{2}$ = 0.70 (CRLR: 466 ms; CRLC: 489 ms; CCLR: 521 ms; CCLC: 492 ms). The data patterns are presented in Fig. 2c.

#### Error rates

In Experiment 3, participants made more errors compared with Experiments 1 and 2. We thus analyzed it as reaction time data. Error rate is the percentage of incorrect probe responses (missed or incorrect response) after correct prime responses. We thus excluded all incorrect prime responses (5.24%).

A 2 (perceptibility: easy vs. hard) × 2 (location relation: repetition vs. change) × 2 (nonspatial feature relation: repetition vs. change) repeated-measures ANOVA on probe error rates revealed a main effect of color response relation, *F*(1, 15) = 19.86, *p* < .001, ${\upeta }_{p}^{2}$ = 0.57 (CR: 3.19%; CC: 5.63%). The interaction of color relation and location relation was significant, *F*(1, 15) = 48.43, *p* < .001, ${\upeta }_{p}^{2}$ = 0.76, depicting a binding pattern: When the color response repeated, participants made less errors when the location repeated (1.05%) compared with changed (5.34%), which was significant when tested against each other, *t*(15) = 5.30, *p* < .001, *d* = 1.32. In contrast, when the color response changed, participants made less errors when the location changed (2.85%) compared with repeated (8.41%), which was significant when tested against each other, *t*(15) = 6.83, *p* < .001, *d* = 1.71. This was not further modulated by perceptibility, *F*(1, 15) = 0.09, *p* = .774, ${\upeta }_{p}^{2}$ = 0.01. No other effects were significant (all *F*s ≤ 2.55, all *p*s ≥ .131).

In the easy perceptibility block, the main effect of color relation was significant, *F*(1, 15) = 22.05, *p* < .001, ${\upeta }_{p}^{2}$ = 0.60 (CR: 2.60%; CC: 5.52%). The main effect of location relation did not reach significance, *F*(1, 15) = 3.41, *p* = .085, ${\upeta }_{p}^{2}$ = 0.19. The interaction of color relation and location relation was significant, *F*(1, 15) = 48.94, *p* < .001, ${\upeta }_{p}^{2}$ = 0.77 (CRLR: 0.72%; CRLC: 4.48%; CCLR: 8.42%; CCLC: 2.61%). In the hard perceptibility block, the main effect of color relation was significant, *F*(1, 15) = 4.63, *p* = .048, ${\upeta }_{p}^{2}$ = 0.24 (CR: 3.78%; CC: 5.74%). The main effect of location relation was not significant, *F*(1, 15) = 0.12, *p* = .737, ${\upeta }_{p}^{2}$ = 0.01. The interaction of color relation and location relation was significant, *F*(1, 15) = 25.28, *p* < .001, ${\upeta }_{p}^{2}$ = 0.63 (CRLR: 1.37%; CRLC: 6.20%; CCLR: 8.40%; CCLC: 3.08%).

### Discussion

In Experiment 3, participants discriminated the color of target dots, with the location feature being irrelevant for responding. We observed strong binding effects between color response and location in both perceptibility conditions. Note that color and response were completely confounded (e.g., Schöpper et al., 2020) and that the resulting data pattern likely resulted from the *response* being bound to the location, as *color* and location often do not bind together (Hilchey et al., 2017; cf. Hommel, 1998, 2007). Although hard perceptibility slowed down overall responding, this did not affect the strength of binding and retrieval. Further, this suggests that targets were discriminable and perceptually dissimilar even in the hard perceptibility condition.

#### Reaction time distribution analyses

Experiments 1 and 2 suggested that slow responses do not lead to retrieval in detection and localization performance. To gain more support for this, we looked at cumulative reaction time distributions based on reaction time percentiles (e.g., Schöpper & Frings, 2022, 2023; Schöpper et al., 2022a, b; Taylor & Ivanoff, 2005). As binding in detection performance can manifest in a main effect with a benefit of (nonspatial) feature repetition (see Schöpper & Frings, 2023; Schöpper et al., 2020), we not only looked at the interaction of color and location, but also at the calculated main effects of color and location.

**Fig. 3:**
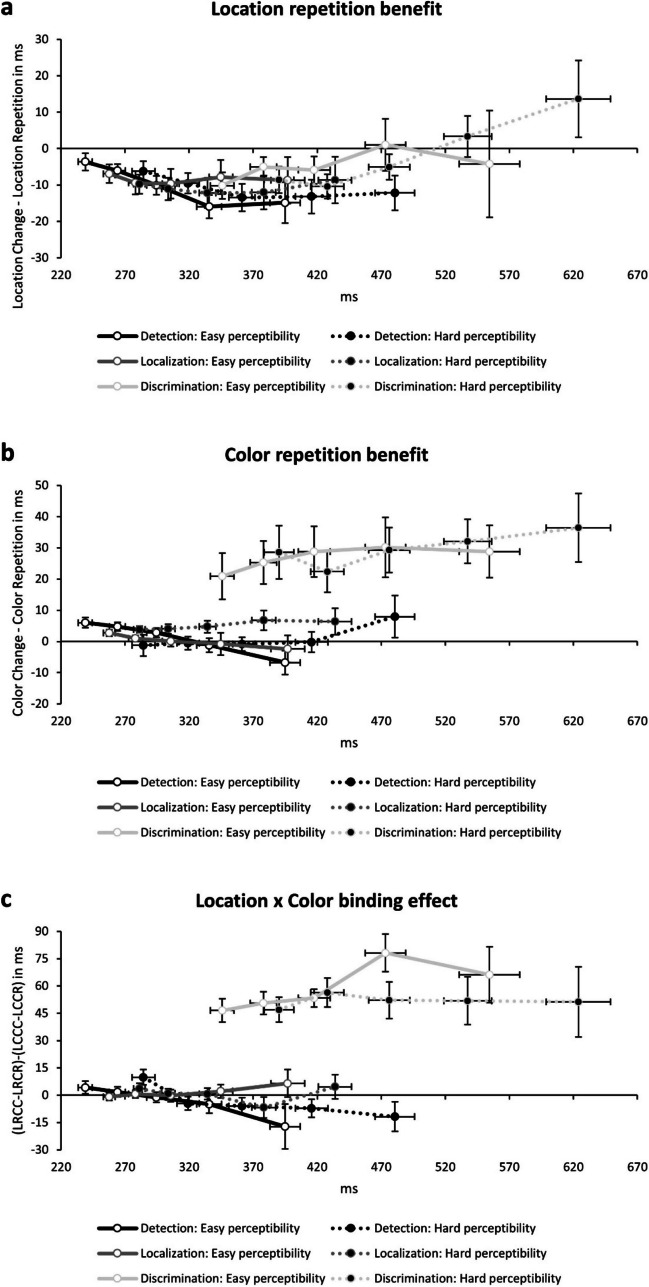
The calculated differential value of **a** the location change benefit (IOR), **b** the color repetition benefit, and **c** the interactions of color and location on the *y*-axis in ms and reaction times on the *x*-axis in ms as a function of percentile (cf. delta plot; De Jong et al., 1994; Ridderinkhof, 2002) and each experiment (Experiment 1: Detection task; Experiment 2: Localization task; Experiment 3: Discrimination task). See main text for explanations. The white (Easy perceptibility) and black (Hard perceptibility) dots represent the 10^th^, 25^th^, 50^th^, 75^th^, and 90^th^ percentile for each function separate for each experiment. Error bars represent standard error of each mean of each averaged percentile for the effect of interest (*y*-axis) and overall reaction time (*x*-axis)

After applying the cut-off criteria mentioned above, we took the 10^th^, 25^th^, 50^th^, 75^th^, and 90^th^ percentile of probe reaction times separate for each participant for each condition in each experiment. We then calculated three differential values for each percentile. First, we calculated IOR as ((LCCR + LCCC)/2) − ((LRCR + LRCC)/2): A negative value resembles a location repetition cost, that is, IOR (see Fig. 3a); for Experiment 2 this additionally resembles a response repetition cost. Second, we calculated ((LRCC + LCCC)/2) − ((LRCR + LCCR)/2), in which a positive value resembles a color repetition benefit (see Fig. 3b); for Experiment 3 this additionally resembles a response repetition benefit. Lastly, we calculated the binding pattern between color and location as (LRCC − LRCR) − (LCCC − LCCR): With this formula the partial repetition costs for location repetition and change are summed up (e.g., Schöpper & Frings, 2022; cf. Singh et al., 2018), indicated by a color repetition benefit over change at location repetitions and a color change benefit over repetition at location changes (see Fig. 3c). To investigate a potential role of response speed we conducted a repeated-measures MANOVA with percentile (10^th^ vs. 25^th^ vs. 50^th^ vs. 75^th^ vs. 90^th^) as the only factor on each calculated effect in each experiment and perceptibility condition.

#### Location repetition cost—IOR

In the easy perceptibility condition in the detection task (Experiment 1), the effect of percentile was significant, *F*(4, 25) = 3.86, *p* = .014, ${\upeta }_{p}^{2}$ = 0.38, suggesting that IOR became stronger with increasing percentile (10^th^: − 4 ms; 25^th^: − 6 ms; 50^th^: − 10 ms; 75^th^: − 16 ms; 90^th^: − 15 ms). In the hard perceptibility condition, the effect of percentile was not significant, *F*(4, 25) = 1.34, *p* = .283, ${\upeta }_{p}^{2}$ = 0.18 (10^th^: − 6 ms; 25^th^: − 10 ms; 50^th^: − 13 ms; 75^th^: − 13 ms; 90^th^: − 12 ms).

In the easy perceptibility condition in the localization task (Experiment 2), the effect of percentile was not significant, *F*(4, 24) = 0.94, *p* = .457, ${\upeta }_{p}^{2}$ = 0.14 (10^th^: − 7 ms; 25^th^: − 9 ms; 50^th^: − 10 ms; 75^th^: − 8 ms; 90^th^: − 9 ms). In the hard perceptibility condition, the effect of percentile was not significant, *F*(4, 24) = 0.69, *p* = .603, ${\upeta }_{p}^{2}$ = 0.10 (10^th^: − 10 ms; 25^th^: − 11 ms; 50^th^: − 12 ms; 75^th^: − 12 ms; 90^th^: − 9 ms).

In the easy perceptibility condition in the discrimination task (Experiment 3), the effect of percentile was not significant, *F*(4, 12) = 1.11, *p* = .395, ${\upeta }_{p}^{2}$ = 0.27 (10^th^: − 10 ms; 25^th^: − 5 ms; 50^th^: − 6 ms; 75^th^: 1 ms; 90^th^: − 4 ms). In the hard perceptibility condition, the effect of percentile was significant, *F*(4, 12) = 4.97, *p* = .013, ${\upeta }_{p}^{2}$ = 0.62, suggesting that IOR became weaker with increasing percentile (10^th^: − 9 ms; 25^th^: − 10 ms; 50^th^: − 5 ms; 75^th^: 3 ms; 90^th^: 14 ms).

#### Color repetition benefit

In the easy perceptibility condition in the detection task (Experiment 1), the effect of percentile was significant, *F*(4, 25) = 2.93, *p* = .041, ${\upeta }_{p}^{2}$ = 0.32, suggesting that an early color repetition benefit disappeared with a tendency to turn into a color change benefit at late percentiles (10^th^: 6 ms; 25^th^: 5 ms; 50^th^: 3 ms; 75^th^: − 1 ms; 90^th^: − 7 ms). In the hard perceptibility condition, the effect of percentile was not significant, *F*(4, 25) = 0.64, *p* = .638, ${\upeta }_{p}^{2}$ = 0.09 (10^th^: − 1 ms; 25^th^: − 1 ms; 50^th^: − 1 ms; 75^th^: 0 ms; 90^th^: 8 ms).

In the easy perceptibility condition in the localization task (Experiment 2), the effect of percentile was not significant, *F*(4, 24) = 0.80, *p* = .537, ${\upeta }_{p}^{2}$ = 0.12 (10^th^: 3 ms; 25^th^: 1 ms; 50^th^: 0 ms; 75^th^: − 1 ms; 90^th^: − 2 ms). In the hard perceptibility condition, the effect of percentile was not significant, *F*(4, 24) = 0.20, *p* = .937, ${\upeta }_{p}^{2}$ = 0.03 (10^th^: 4 ms; 25^th^: 4 ms; 50^th^: 5 ms; 75^th^: 7 ms; 90^th^: 6 ms).

In the easy perceptibility condition in the discrimination task (Experiment 3), the effect of percentile was not significant, *F*(4, 12) = 0.92, *p* = .486, ${\upeta }_{p}^{2}$ = 0.23 (10^th^: 21 ms; 25^th^: 25 ms; 50^th^: 29 ms; 75^th^: 30 ms; 90^th^: 29 ms). In the hard perceptibility condition, the effect of percentile reached significance, *F*(4, 12) = 3.27, *p* = .050, ${\upeta }_{p}^{2}$ = 0.52, suggesting that the color response repetition benefit became slightly stronger with increasing percentile (10^th^: 29 ms; 25^th^: 22 ms; 50^th^: 29 ms; 75^th^: 32 ms; 90^th^: 36 ms).

#### Color–location binding

In the easy perceptibility condition in the detection task (Experiment 1), the effect of percentile was not significant, *F*(4, 25) = 0.94, *p* = .459, ${\upeta }_{p}^{2}$ = 0.13 (10^th^: 4 ms; 25^th^: 2 ms; 50^th^: − 1 ms; 75^th^: − 5 ms; 90^th^: − 17 ms). In the hard perceptibility condition, the effect of percentile was significant, *F*(4, 25) = 4.79, *p* = .005, ${\upeta }_{p}^{2}$ = 0.43, suggesting an interaction congruent with partial repetition costs at the earliest percentile which disappeared with a tendency to turn into an interaction marked by partial repetition benefits at late percentiles (10^th^: 10 ms; 25^th^: − 4 ms; 50^th^: − 6 ms; 75^th^: − 7 ms; 90^th^: − 12 ms).

In the easy perceptibility condition in the localization task (Experiment 2), the effect of percentile was not significant, *F*(4, 24) = 0.42, *p* = .791, ${\upeta }_{p}^{2}$ = 0.07 (10^th^: − 1 ms; 25^th^: 1 ms; 50^th^: 0 ms; 75^th^: 2 ms; 90^th^: 7 ms). In the hard perceptibility condition, the effect of percentile was not significant, *F*(4, 24) = 2.19, *p* = .101, ${\upeta }_{p}^{2}$ = 0.27 (10^th^: 4 ms; 25^th^: 1 ms; 50^th^: 1 ms; 75^th^: − 7 ms; 90^th^: 5 ms).

In the easy perceptibility condition in the discrimination task (Experiment 3), the effect of percentile was not significant, *F*(4, 12) = 2.18, *p* = .133, ${\upeta }_{p}^{2}$ = 0.42 (10^th^: 47 ms; 25^th^: 51 ms; 50^th^: 53 ms; 75^th^: 78 ms; 90^th^: 66 ms). In the hard perceptibility condition, the effect of percentile was not significant, *F*(4, 12) = 1.64, *p* = .229, ${\upeta }_{p}^{2}$ = 0.35 (10^th^: 47 ms; 25^th^: 56 ms; 50^th^: 52 ms; 75^th^: 52 ms; 90^th^: 51 ms).

### Discussion

Analysis of reaction time distributions revealed the following. The IOR effect in the easy perceptibility condition of the detection task (Experiment 1)—the fastest task in the current study—became larger with increasing percentiles. This fits well with IOR taking time to emerge (e.g., Chao et al., 2020; Panis & Schmidt, 2022; Schöpper & Frings, 2023; Taylor & Ivanoff, 2005). Yet the IOR effect in the hard perceptibility condition in Experiment 1 and in both conditions of the localization task (Experiment 2) was stable across percentiles; this suggests a ceiling effect for IOR in our design. In the easy perceptibility condition of the discrimination task (Experiment 3), overall IOR or a significant decrease of it was absent (albeit the pattern being descriptively congruent with IOR occurring in the earliest percentiles). Interestingly, in the hard perceptibility condition, IOR occurred in the earliest percentile but then showed a tendency to turn into a location repetition benefit (cf. Chao, Hsiao, & Huang, 2022). This fits well with IOR being overlapped by co-occurring repetition priming (Klein, 2004) or binding effects (Hilchey et al., 2018; Schöpper & Frings, 2022; Schöpper et al., 2022b) and suggests that fast discrimination responses can be affected by IOR but that this is increasingly masked by late emerging retrieval effects (Schöpper et al., 2022b). Further, this might be interpreted as time courses of IOR and retrieval-based effects being different, with IOR potentially emerging earlier in time than retrieval-based effects (cf. Chao et al., 2020; Chao, Hsiao, & Huang, 2022; Schöpper & Frings, 2022).

A color repetition benefit emerged in the earliest percentiles in the easy perceptibility condition of the detection task, which disappeared in the slower percentiles. Note that this is incongruent with late emerging retrieval but suggests a potential co-occurrence of other effects like priming-of-pop-out (cf. Hilchey et al., 2019; Maljkovic & Nakayama, 1994; Moher & Song, 2014). This is backed up by the overall color repetition benefit found in the hard perceptibility condition in the localization task, which occurred irrespective of response repetitions and changes and remained stable across percentiles. In the other conditions of the detection and localization tasks, no such effects emerged. In the discrimination task, a color response repetition benefit emerged (Frings et al., 2007; Pashler & Baylis, 1991; Schöpper et al., 2022a), with some evidence for an increase in size with increasing percentile in the hard perceptibility condition.

A late emerging interaction of color and location was absent in all conditions of the detection and localization task. Only the hard perceptibility condition of the detection task showed an interaction congruent with partial repetition costs at the earliest percentile which turned into an interaction marked by partial repetition benefits at late percentiles (cf. Schöpper et al., 2022a). Again, this pattern is incongruent with late-emerging partial repetition costs, suggesting a potential interplay with feature repetition benefits in selection performance (cf. Hilchey et al., 2019): For example, it might be beneficial to search for a specific target at a previous location (cf. Krummenacher et al., 2009; Talcott & Gaspelin, 2020). However, this effect then disappeared, showing a tendency for late emerging partial repetition benefits congruent with nonspatial IOR (e.g., Schöpper et al., 2022a). Additionally, due to these potentially co-occurring effects differently affecting fast and slow responses, an overall interaction of location and color was not observed. In the discrimination task the interaction of color response and location was stable across percentiles; because previously increases in binding effects with increasing percentiles have been observed (Chao, Hsiao, & Huang, 2022; Schöpper & Frings, 2022, 2023; Schöpper et al., 2022b), this suggests—as with IOR—a ceiling effect for S-R binding in our design (cf. Schöpper et al., 2022a).

Most crucially, time distributions of all tasks—detection, localization, and discrimination—at least partially overlapped; still, there was no evidence for late emerging retrieval-based effects in the sense of binding and retrieval in action control affecting detection and localization performance.

## General discussion

In the current study participants signaled the detection of target dots (Experiment 1), localized them (Experiment 2), or discriminated their color (Experiment 3). Targets were either red or blue on black background—assumed to be easily perceptible—or light or dark grey on intermediate grey background—thus assumed to be harder to perceive. Participants were slower in responding to grey targets, confirming that perceptibility was impeded in this condition. We observed IOR (e.g., Klein, 2000), that is, a location repetition cost, in both perceptibility blocks of the detection and localization tasks. Yet in detection performance, we neither observed a main effect of color relation nor any interaction between color and location relation. In localization, we observed a benefit of color repetition, which, however, was unaffected by repeating or changing the response. Importantly, this absence of binding and retrieval did not hinge on the perceptibility of targets. In contrast, discriminating the color of targets led to strong binding effects irrespective of perceptibility. Therefore, the current results support the previously proposed task dependency in action control (Schöpper et al., 2020).

The current results replicate that in visual detection (Schöpper & Frings, 2023; Schöpper et al., 2020) and localization (Schöpper & Frings, 2022; Schöpper et al., 2022a) performance binding effects are not observed (see also Huffman et al., 2018). Yet the present results also pinpoint the interpretation of this data pattern. In fact, the explanation of detection and localization responses simply being too fast to be affected by retrieval (e.g., Schöpper et al., 2020) seems unlikely as an explanation for absent binding effects, as participants’ responses were drastically slowed by decreasing the perceptibility of targets. Thus, the horserace assumption of a slow retrieval process versus computing the target response as a sole explanation can be excluded. Rather, explanations based on task-specific components of detection and localization performance like a lack of attention towards target identity (Huffman et al., 2018; cf. Hommel, 2007) or a lack of postselective processing after target identification (Schöpper & Frings, 2022, 2023; Schöpper et al., 2022a, b; see also Hilchey et al., 2020) hold up. Alternatively, both attention and response speed might interplay, in that attention towards targets *and* enough time for retrieval is necessary (cf. Chao, Chen, & Kuo, 2022). This would fit with late emerging binding effects that are eventually absent in (very) fast responding (see Schöpper et al., 2022b). All in all, this corroborates an important limitation for current action control theories in that detection performance, at least when responding with just one manual response (see Chao & Hsiao, 2021; Hilchey et al., 2020, for more complex designs with response/task repetitions and changes) and localization performance seem not to fit into the type of actions that are covered by these frameworks.

To test for time-based explanations, previous research looked at the distribution of reaction times, for example, with Vincentized cumulative reaction time distributions (Ratcliff, 1979) or delta plots (De Jong et al., 1994). By looking at delta plots—that is, plotting the effect of interest in a certain percentile on the y-axis with the mean response speed in each percentile on the *x*-axis (see, e.g., Ridderinkhof, 2002)—evidence for retrieval affecting especially late responses has been found for discriminatory responses (e.g., Schöpper & Frings, 2022; Schöpper et al., 2022b). However, looking at reaction time distributions, it has previously been found that detection responses either lead to nonspatial feature change benefits at location repetitions at very late responses (Chao et al., 2020), or no effects emerge at all (Schöpper & Frings, 2023). Similarly, in localization procedures either feature change benefits (at location repetitions) emerge (Schöpper et al., 2022a) or effects are absent for either fast and slow responses (Schöpper & Frings, 2022). In turn, late responding in previous experiments either showed no impact of nonspatial feature repetition and changes or showed a pattern that fits with nonspatial IOR (e.g., Hu et al., 2011). In the current study we replicated the non-occurrence of binding effects in late detection and localization tasks—also backed-up by reaction time distribution analyses. This allows to conclude that retrieval does either not affect late responding (current study) or affects late responding but incongruent with binding assumptions in action control (for a discussion, see Schöpper & Frings, 2022; Schöpper et al., 2022a).

For greyscale targets in the detection task we did not observe any effects of feature repetition or change, congruent with task-dependent absence of retrieval (Schöpper et al., 2020). However, neither did we observe nonspatial IOR—or larger IOR effects in color repetition compared with color change trials (Chao et al., 2020)—although overall detection performance was slow (cf. Experiment 2 in Chao et al., 2020). In the localization task we found a color repetition benefit in the hard perceptibility block; however, late emerging nonspatial IOR was absent as well (cf. Schöpper et al., 2022a). First, one might muse that different greyscale targets were perceived as too similar, so that nonspatial feature repetitions and changes were not perceived as such. Contrary, in such a case one might muse it would be especially useful for the cognitive system to retrieve a previous target because this would ease detection on an intermediate-colored background; this would be in line with feature repetition benefits in visual search performance (e.g., Moher & Song, 2014), for which we found some evidence in localization data. Further, the results of the discrimination task (Experiment 3) suggest that even if hard to perceive, targets were still discriminable. Second, it is possible that nonspatial features affected responding but that the underlying processes balanced each other out: On one hand a detection cost favoring a nonspatial feature *change benefit* (Chao et al., 2020; Hu et al., 2011; Lupiáñez et al., 2013), on the other hand retrieval favoring a nonspatial feature *repetition benefit* (caused by binding of response and nonspatial feature as in auditory detection performance; Schöpper & Frings, 2023). In turn, no effect was observed because both effects worked against each other (for this argument, see Schöpper & Frings, 2022). Third, nonspatial IOR effects seem to be not as robust as location-based IOR effects (e.g., Chao et al., 2020; Chao, Hsiao, & Huang, 2022; see also Kwak & Egeth, 1992); in turn, retrieval-based effects in the realm of S-R binding might have been absent due to task demands, whereas nonspatial IOR effects were absent due to so far unclear components of the experimental design.

Recently, S-R binding in detection performance has been found when the spatial position of the target has to be processed for giving the detection response (Hilchey et al., 2020), when the detection response is a cued left/right response executed upon target detection (Chao & Hsiao, 2021; Chao, Hsiao, & Huang, 2022), or when the target is presented auditorily (Mondor & Leboe, 2008; Schöpper & Frings, 2023). However, if the detection response is simply operationalized as the pressing of the same key for every onsetting visual target (cf. Huffman et al., 2018), S-R binding effects are absent (Huffman et al., 2018; Schöpper et al., 2020). Future studies could investigate if this task-dependent absence occurs during binding or retrieval or both. Effects of nonspatial IOR suggest that integration of stimulus information is possible, which, however, leads to different data patterns when retrieved/processed (cf. Chao et al., 2020; Schöpper et al., 2022a).

## Conclusion

Slowing down response speed by making targets hard to perceive did not lead to the observation of retrieval in visual detection and localization performance. This replicates Schöpper et al. (2020) and Schöpper and Frings (2022) in that binding effects are task dependent and shows that this task dependency occurs irrespective of response speed.
